# Preventive Effects of Green Tea Extract against Obesity Development in Zebrafish

**DOI:** 10.3390/molecules26092627

**Published:** 2021-04-30

**Authors:** Liqing Zang, Yasuhito Shimada, Hiroko Nakayama, Hirotaka Katsuzaki, Youngil Kim, Djong-Chi Chu, Lekh Raj Juneja, Junya Kuroyanagi, Norihiro Nishimura

**Affiliations:** 1 Graduate School of Regional Innovation Studies, Mie University, Tsu, Mie 514-8507, Japan; 27293301@m.mie-u.ac.jp (H.N.); nishimura.norihiro@mie-u.ac.jp (N.N.); 2 Zebrafish Drug Screening Center, Mie University, Tsu, Mie 514-8507, Japan; shimada.yasuhito@mie-u.ac.jp; 3 Department of Integrative Pharmacology, Graduate School of Medicine, Mie University, Tsu, Mie 514-8507, Japan; 4 Department of Bioinformatics, Advanced Science Research Promotion Center, Mie University, Tsu, Mie 514-8507, Japan; 5 Department of Life Sciences, Graduate School of Bioresources, Mie University, Tsu, Mie 514-8507, Japan; katsuzak.bio@mie-u.ac.jp; 6 Rohto Pharmaceutical Co., Ltd., Osaka 530-0011, Japan; youngil-kim@pharmafoods.co.jp (Y.K.); ssj3590@yahoo.co.jp (D.-C.C.); juneja@rohto.co.jp (L.R.J.); 7 UMOU Science Lab, Matsusaka, Mie 515-0303, Japan; kuro@umou.or.jp

**Keywords:** green tea extract, Mibyo, visceral adipose tissue, obesity, zebrafish, RNA-seq

## Abstract

Various natural products (NPs) have been used to treat obesity and related diseases. However, the best way to fight obesity is preventive, with accurate body weight management through exercise, diet, or bioactive NPs to avoid obesity development. We demonstrated that green tea extract (GTE) is an anti-obesity NP using a zebrafish obesity model. Based on a hypothesis that GTE can prevent obesity, the objective of this study was to assess GTE’s ability to attenuate obesity development. Juvenile zebrafish were pretreated with GTE for seven days before obesity induction via a high-fat diet; adult zebrafish were pretreated with GTE for two weeks before obesity induction by overfeeding. As a preventive intervention, GTE significantly decreased visceral adipose tissue accumulation in juveniles and ameliorated visceral adiposity and plasma triglyceride levels in adult zebrafish obesity models. RNA sequencing analysis was performed using liver tissues from adult obese zebrafish, with or without GTE administration, to investigate the underlying molecular mechanism. Transcriptome analysis revealed that preventive GTE treatment affects several pathways associated with anti-obesity regulation, including activation of STAT and downregulation of CEBP signaling pathways. In conclusion, GTE could be used as a preventive agent against obesity.

## 1. Introduction

Overweight and obesity have become global human health issues in recent years. More than 1.9 billion adults have been diagnosed as overweight; of these, >650 million are obese [1]. Overweight and obesity are defined as abnormal or excessive fat accumulation, and obesity has been recognized as a disease since 2013 [2]. Frequent health-related consequences associated with obesity include cardiovascular diseases, diabetes, musculoskeletal disorders, and cancer [1]. Apart from the aging-related reduction in metabolism, lifestyle-related nutritional and metabolic disorders are major risk factors for obesity. Numerous natural products (NPs) have been found to ameliorate obesity and related metabolic diseases, including plant-derived NPs (e.g., Panax ginseng, Citrus limon, Palmaria mollis) and animal-derived NPs (e.g., chitosan from crab and shrimp shells and fish oil) [3,4,5,6,7]. However, most importantly, individuals should aim to avoid developing obesity throughout life, since drastic weight change is a significant burden on the body. Lifestyle changes, such as increased physical activity, decreased calorie intake, and consumption of particular bioactive NPs, have effectively helped individuals to prevent obesity [8].

Oriental medicine defines the body’s condition as existing in three states: healthy state, predisease state, and disease state. The predisease state, also termed Mibyo in traditional Japanese medicine or Wei Bing in traditional Chinese medicine, is a disease-oriented state that can easily become a disease if no cure is applied [9]. In Oriental medicine, predisease treatment has long been proposed as the best therapeutic strategy to prevent illness. In brief, both preventive treatment before the occurrence of a disease and treatment during the predisease state remain critical for maintaining or improving human health. Several NPs or traditional medicine formulas reportedly possess preventive effects or can be used to treat predisease states [10,11,12].

Green tea is a well-known functional material rich in polyphenols, known as catechins [13,14]. The major catechin in green tea, (-)-epigallocatechin-3-gallate (EGCG), is considered a principal contributor to the health benefits of green tea, including anticancer [15,16,17], antimetabolic syndrome [18,19,20], antiviral [21], and anti-infectious effects [22]; cardiovascular protective effects [23]; and neuroprotective effects [24]. In 2019, green tea extract (GTE) was reported to reduce visceral adipose tissue (VAT) volume and decrease plasma triglyceride (TG) and total cholesterol (TCHO) levels in diet-induced obesity models in zebrafish [25]. In several epidemiological analyses, individuals who regularly drink green tea were found to be less obese [19,26,27,28]. We hypothesized that GTE has the potential to prevent obesity. Accordingly, a preventive intervention with GTE was performed using juvenile and adult zebrafish with diet-induced obesity. RNA sequencing (RNA-seq) of liver tissues from adult zebrafish was also performed to identify the molecular mechanisms underlying the preventive anti-obesity effects of GTE.

## 2. Results

### 2.1. Preventive GTE Administration Decreased VAT Volume in Juvenile Zebrafish

**Figure 1:**
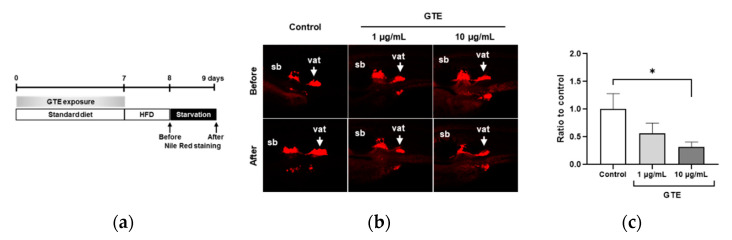
Preventive administration of green tea extract (GTE) suppresses visceral adipose tissue (VAT) accumulation in juvenile zebrafish. (**a**) Scheme of the experimental design. (**b**) Representative images from live zebrafish juveniles after performing two Nile red staining processes shown in (**a**). vat, visceral adipose tissue (VAT); sb, swim bladder; control, HFD-treated control juvenile. (**c**) Quantification of the intensity of the Nile-red-signal-positive area. The *y* axis shows the ratio of two Nile red staining processes performed compared with the control group. * *p* < 0.05 vs. control, *n* = 5. Error bars indicate the standard deviation (SD).

HPLC analysis was performed to identify the components of GTE; the chromatograms are shown in Supplementary Figure S1. Four major components, (-)-epigallocatechin gallate (EGCG), (-)-epicatechin gallate (ECG), (-)-epicatechin (EC), and (-)-epigallocatechin (EGC), were detected, accounting for 70.3%, 16.3%, 8.8%, and 2.4%, respectively. To assess the preventive effect of GTE against obesity development, normal healthy juveniles were pretreated with GTE for seven days (Figure 1a), then administration was discontinued, and a zebrafish obesogenic test (ZOT) was performed [29]. Nile red fluorescence was used as a probe to visualize and quantify lipid localization and accumulation, and staining was performed immediately after high-fat diet (HFD) feeding and after one day of starvation to quantify changes in the VAT volume. In zebrafish juveniles, a low dose of GTE (1 μg/mL) tended to decrease the VAT volume, whereas a higher GTE dose (10 μg/mL) significantly reduced VAT accumulation when compared with the HFD control (32%, *p* < 0.05; Figure 1b,c).

### 2.2. Preventive GTE Treatment Reduced VAT and Plasma TG Levels in Adult Obese Zebrafish

**Figure 2:**
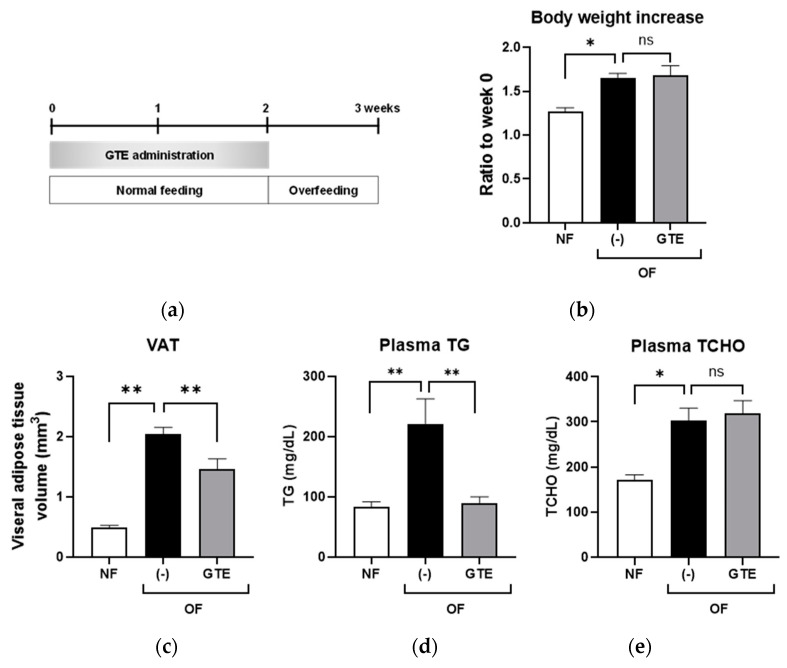
Preventive administration of green tea extract (GTE) reduces VAT volume and decreases plasma triglyceride (TG) levels in adult obese zebrafish. (**a**) Experimental design scheme using adult zebrafish, (**b**) change in body weight, (**c**) change in VAT volume measured by micro-CT, (**d**) change in plasma TG and (**e**) TCHO levels. * *p* < 0.05, ** *p* < 0.01, *n* = 5. Error bars indicate the standard deviation (SD). VAT, visceral adipose tissue; micro-CT, micro-computed tomography; TCHO, total cholesterol.

GTE was administered orally to adult zebrafish. In the present study, GTE was administered to adult fish for the first two weeks with a regular diet and then discontinued during the third week of overfeeding with *Artemia* for obesity induction. A schematic of the experimental design is presented in Figure 2a. Preventive GTE treatment did not affect body weight loss (Figure 2b). However, three-dimensional micro-computed tomography (3D micro-CT) analysis revealed that the GTE group exhibited a significantly decreased VAT volume when compared with the overfeeding (OF) group (*p* < 0.05; Figure 2c). The fasting plasma concentrations of TG and TCHO were assessed to investigate the effect of GTE on plasma lipoproteins. Plasma TG levels were significantly higher in the OF group (221 ± 41 mg/dL) than in the normal feeding (NF) group (82 ± 9 mg/dL; *p* < 0.001), whereas GTE treatment significantly decreased TG levels when compared to the OF group (90 ± 10 mg/dL; *p* < 0.01) (Figure 2d). However, GTE did not affect the plasma TCHO levels (Figure 2e).

### 2.3. Molecular Mechanisms Underlying the Preventive Anti-Obesity Effects of GTE

**Figure 3:**
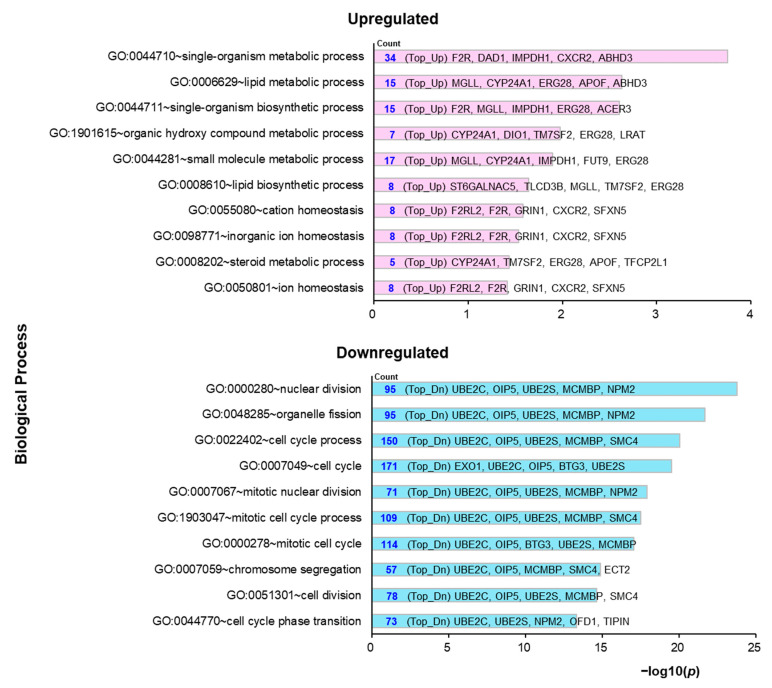
GO pathway analysis for preventive administration of GTE compared with OF. The top 10 enriched GO functions for upregulated (Top_Up, pink) and downregulated (Top_Dn, blue) are shown in the −log10 *p*-value (−log10(*p*)). GO, Gene Ontology; GTE, green tea extract; OF, overfeeding.

To elucidate the molecular mechanisms underlying the preventive anti-obesity ability of GTE, RNA sequencing (RNA-seq)-based transcriptomic analysis of liver tissues derived from adult obese zebrafish treated with or without GTE was performed. Among the 25,281 sequenced *Danio rerio* genes, 13,625 genes were converted to human orthologs. A total of 1115 differentially expressed genes (DEGs) were identified, which were significantly upregulated or downregulated following preventive GTE administration when compared with OF (fold-change > 2 and <0.5; *p* < 0.05; Supplementary Table S1). Using these DEGs, a Gene Ontology (GO) analysis using the free tool Database for Annotation, Visualization and Integrated Discovery (DAVID) 6.8 (https://david.ncifcrf.gov/summary.jsp; accessed on 7 February 2021) was performed according to the protocol by Huang et al. [30]. Of the 1114 input genes, 1039 (93%) were present in the DAVID GO dataset. The three main GO categories with the following DAVID tools were analyzed: biological process (BP_ALL), cellular component (CC_ALL), and molecular function (MF_ALL). The results are shown in Supplementary Table S2. The GO terms in the BP_ALL category by separately inputting the upregulated and downregulated DEGs are shown in Figure 3. For upregulated genes, the top GO terms were related to metabolic processes, including lipid metabolic processes, biosynthetic processes, and small-molecule metabolic processes. Several downregulated genes were associated with cellular processes, including nuclear division, cell cycle, and cell division. The biological pathways regulated by the preventive administration of GTE were further investigated, and the top 15 mapped pathways are listed in Table 1 (*p* < 0.05). There were several significantly regulated pathways related to transcription and cellular processes, such as transcription regulation by RB/2F, that contribute to cellular metabolic responses.

### 2.4. Comparison of Mechanisms Underlying Preventive Anti-Obesity Effects and Anti-Obesity Effects of GTE

**Figure 4:**
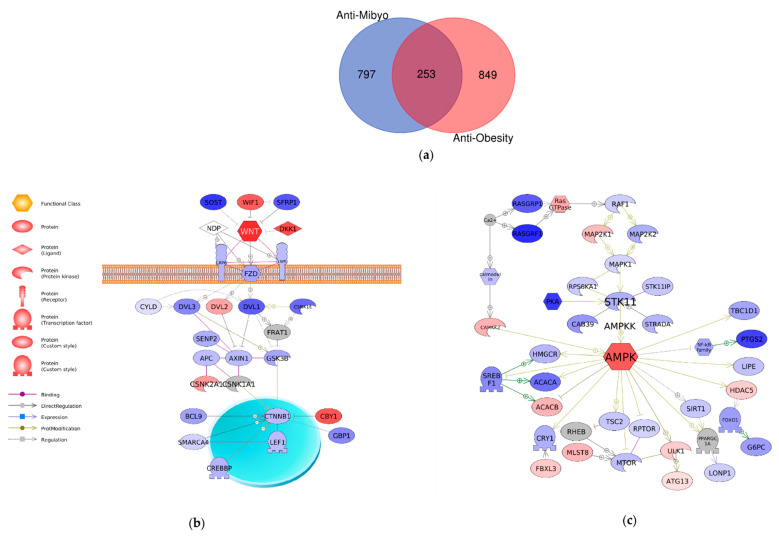
Comparison of the two datasets. (**a**) A Venn diagram comparing differentially expressed genes in anti-Mibyo and anti-Obesity datasets. (**b**) Wnt canonical signaling and (**c**) AMPK signaling were identified using altered genes in the anti-Mibyo dataset. The red and blue colors indicate genes with upregulated and downregulate expression, respectively. Gray denotes genes that were not detected or statistically cut off in the RNA-seq assay.

The anti-obesity effects of GTE have been evaluated, and the underlying molecular mechanisms might contribute to the activation of the Wnt/β-catenin and adenosine monophosphate-activated protein kinase (AMPK) signaling pathways [25]. Herein, the present and previous transcriptome analyses were compared to clarify the molecular mechanisms unique to the preventive anti-obesity effects of GTE. To distinguish the two different datasets, the previous dataset was named anti-Obesity and the present dataset was named anti-Mibyo. First, common significant DEGs in the two datasets were extracted. A total of 253 genes were identified as common GTE-regulated genes in the two studies (Figure 4a). Notably, the Wnt and AMPK signaling pathways were also activated by preventive GTE administration (Table 1), which is a common GTE-regulated pathway regardless of the conditions for administration (Figure 4b,c).

**Figure 5:**
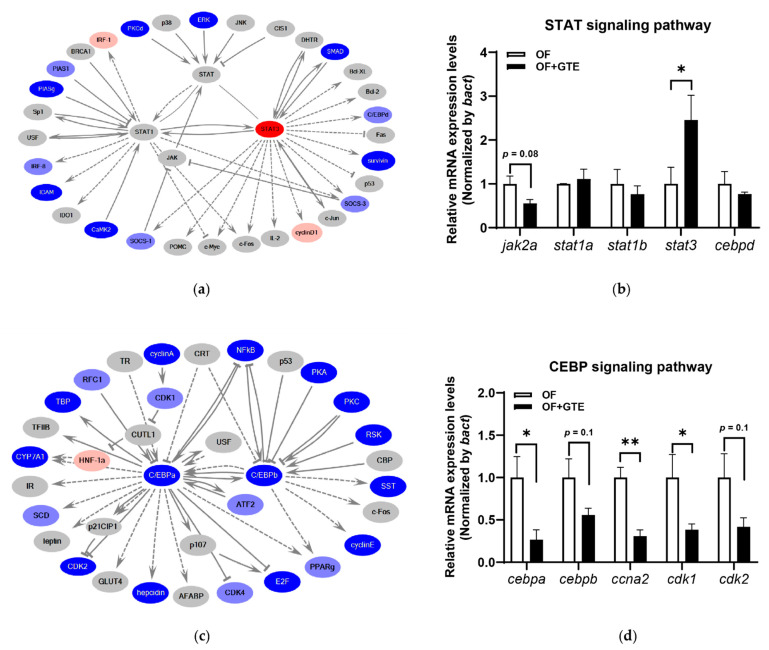
The supposed molecular pathways in the altered gene expression in the anti-Mibyo study. (**a**) Molecular network of transcription regulation of STAT, (**b**) selected gene expression level changes in the STAT signaling pathway, (**c**) molecular network of transcription regulation of CEBP, and (**d**) selected gene expression level changes in the CEBP signaling pathway. The red and blue colors indicate upregulated and downregulated molecules, respectively. Gray denotes genes that were not detected or statistically cut off in the RNA-seq assay. * *p* < 0.05, ** *p* < 0.01 vs. OF group, *n* = 4. Error bars indicate the standard deviation (SD). STAT, signal transducer and activator of transcription; CEBP, CCAAT enhancer-binding protein.

Next, GO and pathway analyses were performed using the 797 genes that were statistically regulated in the anti-Mibyo but not in the anti-Obesity study. GO analysis revealed that preventive GTE administration mainly exhibited positive regulation effects on the cellular process, biological process, and metabolic process (Table 2). Then, an in silico molecular network analysis was performed to predict transcriptional regulators. Among the top 15 pathways, transcriptional regulation by STAT and transcriptional regulation by CEBP had the highest scores (Table 3). The details of these two signaling pathway networks are shown in Figure 5. The key genes of the STAT signaling pathway, signal transduction, and activation of transcription 3 (STAT3) were upregulated by GTE (Figure 5a). The relative mRNA expression levels of the selected genes in this pathway were validated by qPCR analysis (Figure 5b). *Janus kinase 2a* (encoding a homolog of mammalian JAK2) showed a trend toward a decrease (*p* = 0.08), and *stat1a* and *stat1b* were not significantly altered by GTE administration. However, the expression level of *stat3* was significantly upregulated by GTE (*p* < 0.05). Furthermore, GTE inhibited the CCAAT enhancer-binding protein (CEBP) signaling pathway (Figure 5c). Quantitative real-time PCR revealed that the mRNA expression level of *cebpa* was significantly downregulated (*p* < 0.05), and *cebpb* was downregulated by GTE (*p* = 0.1). Other CEBPA-related genes, cyclin a2 (*ccna2*), cyclin-dependent kinase 1 (*cdk1*), and *cdk2*, were all downregulated by GTE administration (*p* < 0.01, *p* < 0.05, and *p* = 0.1, respectively; Figure 5d).

## 3. Discussion

In the present study, we proposed a new model using diet-induced obesity in zebrafish, called Mibyo. This strategy enabled the identification of compounds that could prevent the development of obesity by pre-administration. In this study, we pre-administered GTE and elucidated its preventive effects by determining the transcriptome mechanisms.

Green tea is a bioactive NP that is used to improve human health. The attractive health benefits can be divided into two categories: (1) therapeutic effects (consumption of green tea or catechins can improve parameters associated with various diseases) [14,31,32] and (2) preventive activities (preventive intervention with green tea or catechins may prevent the onset or progression of a particular disease, including cancers, cardiovascular disease, and cognitive dysfunction) [17,19,33,34]. As green tea is popular as a beverage and in diets, it can potentially act as an anti-Mibyo material, which aims to improve the stability of health conditions and prevent the development of one or several diseases in daily life. In the present study, the effects of GTE on obesity development were assessed. We developed a novel protocol that first administered GTE to zebrafish for a sufficiently prolonged period to mimic a daily consumption lifestyle and then discontinued GTE administration to induce obesity. Surprisingly, the preventive intervention of GTE suppressed VAT accumulation in juvenile and adult obesity zebrafish models and decreased plasma TG levels in adult obese zebrafish. After a specific period of GTE consumption, individuals resisted overfeeding stimulation, although GTE intake was stopped. To the best of our knowledge, this is the first study to demonstrate the preventive and continuous anti-obesity effects of GTE, which suggests that GTE is an important anti-Mibyo material for human health.

To investigate the underlying molecular mechanisms of the preventive anti-obesity effects of GTE, transcriptome analysis was performed using liver tissues of obese zebrafish treated with or without GTE. The GO term analysis revealed that GTE regulated the core cellular functions, such as metabolic processes and cellular processes, in the molecular network via upregulated or downregulated target genes (Figure 3, Table 2). It is well known that the development and progression of obesity involve numerous molecular and cellular events that are influenced by genetic and environmental factors. Various cellular events have been demonstrated to cause or inhibit obesity, including cellular signaling pathways related to oxidative stress, lipid metabolism, and mitochondrial β-oxidation, which may lead to inflammation, adipocyte differentiation, cell death, lipogenesis, and lipolysis [35,36]. The lipid metabolic process (GO: 0006629; synonyms: lipid metabolism) was significantly enriched in genes upregulated by GTE, indicating that GTE may ameliorate lipid dysmetabolism in the liver and substantially impact adipocytes. The ubiquitin-conjugating enzyme E2 C (UBE2C) was the most abundant gene in the top 10 GO terms significantly enriched in the downregulated genes by GTE (Table 2). UBE2C is required for the destruction of mitotic cyclins and cell cycle progression and is involved in cancer progression [37,38]. Although there have been no reports on the relationship between UBE2C and obesity, we suppose that UBE2C might be a novel target of GTE, and further study is needed to confirm this hypothesis. To identify the specific cellular signaling pathways regulated by GTE, pathway analysis was performed, and the top 15 regulated signaling pathways with the smallest *p*-value are shown in Table 1. The cell cycle regulators, retinoblastoma/transcription factor E2F (RB/E2F) complexes, and cyclin-dependent kinases (CDKs) were positively regulated by preventive GTE administration. These cellular transcription factors have been shown to contribute to metabolic responses, such as cell growth, development, and proliferation. Dysregulation of the cyclin–CDK–Rb–E2F1 pathway may lead to metabolic perturbations and metabolic diseases, including obesity and diabetes [39]. We hypothesize that the preventive anti-obesity effects of GTE may partially contribute to the regulation of the RB/E2F and CDK signaling pathways. Additionally, GTE can suppress lipid accumulation in obese zebrafish by activating the Wnt and AMPK signaling pathways [25]. These two pathways were also identified as target pathways in the anti-Mibyo effects of GTE (Table 1, Figure 4), which may be part of the molecular mechanisms underlying the resistance of individuals to overfeeding stimulation even when GTE intake has been discontinued.

Furthermore, a comparative transcriptome analysis between a preventive anti-obesity (anti-Mibyo) study and an anti-obesity study of GTE in obese zebrafish was performed to determine pathways unique to the anti-Mibyo study. We focused on the top two STAT and CEBP signaling pathways among the extracted original signaling pathways (Table 3). The JAK/STAT signaling pathway regulates several cytokines, growth factors, and hormones related to adipocyte development and function [40,41]. A previous report suggested that STAT3 can promote lipolysis and inhibit adipogenesis in a mouse model [42], and STAT3 activation can prevent hepatosteatosis and HFD-induced hepatic fat accumulation [43,44]. In our study, *stat3* was significantly upregulated by preventive GTE treatment in the qPCR validation study (Figure 5b). Although the effects of GTE on hepatosteatosis were not examined, we hypothesize that GTE exerts VAT- and plasma TG-reducing effects via the activation of STAT3 and its related gene networks. Additionally, CEBPs are key regulators of adipocyte differentiation. CEBPB and CEBPD are considered to activate adipogenic genes, such as PPARγ and CEBPA, to differentiate into mature adipocytes [45]. Negative regulation of these factors is expected to prevent adipocyte differentiation and further interfere with obesity [46]. Preventive GTE treatment downregulated the mRNA expression of CEBPA and CEBPB (Figure 5c,d). This result is consistent with previous reports demonstrating that catechins, particularly EGCG, can inhibit preadipocyte differentiation, decrease adipocyte proliferation, and induce adipocyte apoptosis partially via the CEBPs/PPARγ pathways [47,48]. Moreover, Wu et al. reported that EGCG induces the apoptosis of 3T3-L1 preadipocytes by decreasing Cdk2 activity [49]. Downregulated cdk2 expression levels were also observed after preventive GTE treatment. Therefore, we conclude that the visceral adipocytic suppressive effects of preventive GTE administration may partially occur via suppression of the CEBP signaling pathway.

The limitation of this study is that green tea extract, a mixture of several bioactive molecules, was used to assess the functions. The main component of GTE is EGCG, present at more than 70%, suggesting that EGCG may be the responsible biomolecule exerting preventive and anti-obesity effects of GTE. However, further studies are needed to confirm this hypothesis. We will administer EGCG alone to determine whether it shows a similar preventative property or acts in synergy with other polyphenols, especially catechins.

In summary, we found that preventive GTE administration significantly decreases VAT accumulation in zebrafish obesity models. Transcriptome analysis revealed that GTE exerts preventive anti-obesity effects partially by activating the STAT and downregulating the CEBP signaling pathways, the unique signaling pathways in the anti-Mibyo study. Our results suggest that GTE can be used as a potential agent to prevent human obesity and related metabolic disorders and could be helpful for the development of anti-obesity medicines. However, further studies are needed to confirm this hypothesis; other related processes and pathways must be validated. The results need to be confirmed in other animal models, and safe and effective doses must be determined before human clinical trials in normal or metabolic syndrome high-risk populations could be initiated.

## 4. Materials and Methods

### 4.1. Ethics Statement

All animal procedures were performed according to Japan’s Act on Welfare and Management of Animals (Ministry of Environment of Japan) and complied with international guidelines. Ethical approval from the local Institutional Animal Care and Use Committee was not sought, as this law does not mandate the protection of fish.

### 4.2. Zebrafish Husbandry

Wile-type AB strain zebrafish (ZFIN: Zebrafish International Research Center, Eugene, OR, USA) were handled and maintained according to standard ZFIN protocols (https://wiki.zfin.org/display/prot/ZFIN+Protocol+Wiki; accessed on 26 April 2020). Juvenile zebrafish (30–89 days postfertilization (dpf)) and adult zebrafish (>90 dpf) were maintained in a zebrafish cultivation system with a photoperiod of 14:10 h (light:dark). The fish were fed GEMMA Micro 75, 150, and 300 (Skretting, Fontaine-les-Vervins, France) based on their developmental stage and length.

### 4.3. Chemicals

Commercially available standardized GTE (PF-TP80) was purchased from Pharma Foods International Co., Ltd. (Kyoto, Japan). For the preparation of GTE, in brief, green tea leaves (*Camellia sinensis* (L.) O. Kuntze) were immersed in hot water (80–85 °C) for 10 min. After filtration and concentration under vacuum, the same amount of ethyl acetate was added and mixed. The ethyl acetate phase was harvested, and GTE was obtained by concentrating the solution, spray drying, and sieving. In this study, GTE was mixed with distilled water and vortexed to prepare a 100 mg/mL stock solution.

### 4.4. Zebrafish Obesogenic Test (ZOT)

ZOT was performed as previously described, with some modifications [25]. The experimental design is illustrated in Figure 1a. Briefly, zebrafish juveniles with a standard length of approximately 10 mm were selected and divided into three groups: control group, GTE 1 μg/mL group, and GTE 10 μg/mL group. Each group was composed of five fish and placed into one well of a 6-well plate filled with 5 mL of 0.3× Danieau’s solution (17.4 mM NaCl, 0.21 mM KCl, 0.12 mM MgSO_4_, 0.18 mM Ca(NO_3_)_2_, and 1.5 mM 4-(2-hydroxyethyl)-1-piperazinyl-ethane-2-sulfonic acid (HEPES); pH 7.6). Working solutions of GTE (1 and 10 μg/mL) were prepared by diluting the stock solution with 0.3× Danieau’s solution.

During the first 7 days of this experiment, juveniles were exposed to GTE working solutions and fed a standard diet (Gemma Micro 150). The solution in each well was changed daily. At 8–9 days (ZOT) of the experiment, the two doses of GTE were discontinued. On the first day of ZOT, juveniles were fed 0.1% hard-boiled chicken egg yolk twice a day as the HFD. During HFD feeding, the plate was shaken at 150 rpm, and a final concentration of 10 µg/mL of kanamycin (Nacalai Tesque, Kyoto, Japan) was added to each well to protect the juveniles from bacteria. On the second day, all fish were starved, and the volume of VAT was measured before and after starvation by Nile Red staining. For Nile Red staining, Nile Red (Tokyo Chemical Industry, Tokyo, Japan) stock solution (0.5 mg/mL) was prepared in acetone. The working solution (5 μg/mL) was obtained by diluting the stock solution in 0.3× Danieau’s solution immediately before use. Fish were exposed to the Nile Red working solution in the dark for 30 min at 28 °C and washed twice with water for 5 min. Fish were then anesthetized in 500 ppm 2-phenoxyethanol (Wako Pure Chemicals, Osaka, Japan), and fluorescence images were obtained using a BZ-X710 fluorescence microscope (Keyence, Tokyo, Japan). Quantification of Nile-Red-positive signals was performed using ImageJ software (Fiji distribution, v.1.52p; National Institutes of Health, Bethesda, MD, USA).

### 4.5. Preventive GTE Administration to Adult Zebrafish

As previously described, 10% GTE-containing zebrafish food was orally administered to adult zebrafish [50]. Female adult zebrafish were randomly assigned to three groups with five fish/2 L tanks: (1) NF group—the zebrafish were fed a normal diet (gluten granules) throughout the experimental period (3 weeks) and additionally fed 5 mg of cysts/fish/day of *Artemia* (brine shrimp) in the third week; (2) overfeeding group—the zebrafish were fed a normal diet (gluten granules) for 3 weeks and overfed with 60 mg of cysts/fish/day of Artemia [51] in the third week; and (3) GTE group—the zebrafish were fed GTE-containing food (250 µg/g body weight/day) for the first 2 weeks and subsequently overfed during the third week. During feeding, the tank water flow was stopped for 2 h. The remaining *Artemia* was removed once daily by vacuuming to avoid water pollution. Body length and weight were measured weekly. Blood glucose, plasma TG, and plasma TCHO levels were measured at the end of the experiment [52,53]. A 3D micro-CT scan was performed using an in vivo System R mCT 3D micro-CT scanner (Rigaku, Tokyo, Japan), and the VAT volume was measured as previously described [25].

### 4.6. Library Construction and High-Throughput Sequencing

The livers of adult zebrafish were isolated, and total RNA was extracted using TRIzol reagent (Life Technologies, Carlsbad, CA, USA) and the QIAGEN RNeasy Mini-prep Kit (Qiagen, Hilden, Germany) [54]. DNase digestion was performed on the column membranes to eliminate DNA contamination (RNase-Free DNase Set; Qiagen). The content and quality of the total RNA were measured using a Qubit 4 Fluorometer (Thermo Fisher Scientific, Waltham, MA, USA) and an Agilent 2100 Bioanalyzer System using the RNA 6000 Nano LabChip^®^ kit (Agilent Technologies, Santa Clara, CA, USA), respectively. Ribosomal RNA was depleted using the RiboMinus™ Eukaryote System v2 kit (Thermo Fisher Scientific) according to the manufacturer’s protocol. The integrity of the rRNA-depleted RNA was further confirmed using an Agilent 2100 Bioanalyzer System (Agilent Technologies). Only samples with an RNA integrity number (RIN) of >8 underwent the next step according to the manufacturer’s protocol. Finally, four samples from each group were used for RNA library construction using the Ion Total RNA-Seq Kit v2 (Life Technologies, Carlsbad, CA, USA) according to the manufacturer’s instructions. Preparations containing bar-coded libraries were loaded into 318 chips and sequenced on an Ion PGM system (Life Technologies). Signal processing and base calling were performed using Torrent Suite software version 4.0.1. Adapter sequences were trimmed using the same software.

### 4.7. Bioinformatics Analysis

Bioinformatics analysis was performed using CLC Genomics Workbench 20.0.2 (Qiagen Bioinformatics, Germantown, MD, USA). The raw reads were cleaned by trimming low-quality sequences with quality scores of <13. The data were then mapped to the annotated *Danio rerio* genome to build GRCz10. The expression of each transcript was detected and normalized using the trimmed mean of M values (TMM) method, followed by a per-sample 75% upper quartile (UQ) [55]. After statistical tests, DEGs were identified (fold-change > 2 and < 0.5; *p* < 0.05). Molecular network and pathway analyses were performed using KeyMolnet software (KM Data, Tokyo, Japan) [56].

### 4.8. Real-Time Quantitative PCR (qPCR)

cDNA from 500 ng of total RNA was synthesized using the ReverTra Ace qPCR RT Kit (Toyobo, Osaka, Japan). qPCR analysis was performed using the Power SYBR Green Master Mix (Applied Biosystems, Foster City, CA, USA) and the ABI StepOnePlus Real-Time PCR System (Applied Biosystems) according to the manufacturer’s instructions. Relative mRNA expression levels were normalized using *bact*. Primer sequences are listed in Supplementary Table S3.

### 4.9. Statistical Analysis

Data were analyzed using Student’s *t*-test or one-way analysis of variance (ANOVA) with the Bonferroni–Dunn multiple comparison tests, depending on the number of comparisons. Statistical analyses were performed using GraphPad Prism version 9.0.1 (GraphPad Software Inc., San Diego, CA, USA). All results are presented as the mean ± SD. Statistical significance was set at *p* < 0.05.

## Figures and Tables

**Table 1:** The top 15 pathways were regulated by preventive administration of GTE in obese zebrafish (*p* < 0.05).

Pathway	*p*-Value
Transcriptional regulation by RB/E2F	2.84 × 10^−13^
Condensin signaling pathway	1.37 × 10^−9^
Calcium signaling pathway	2.90 × 10^−9^
Aurora kinase signaling pathway	5.38 × 10^−6^
Cell cycle	6.33 × 10^−6^
HAT signaling pathway	1.03 × 10^−5^
Transcriptional regulation by FOXM	3.01 × 10^−5^
CDK signaling pathway	3.02 × 10^−5^
ING signaling pathway	9.52 × 10^−5^
PLK signaling pathway	1.08 × 10^−4^
Wnt signaling pathway	3.32 × 10^−4^
Gene regulation by CPEB	4.27 × 10^−4^
Kinesin family signaling pathway	4.94 × 10^−4^
Hedgehog signaling pathway	9.42 × 10^−4^
AMPK signaling pathway	2.36 × 10^−2^

**Table 2:** The top 10 GO terms were specifically regulated by GTE’s preventive administration in obese zebrafish (*p* < 0.05).

Function Name	GO ID	Count	*p*-Value
Positive regulation of cellular process	GO:0048522	69	6.63 × 10^−16^
Positive regulation of biological process	GO:0048518	72	8.88 × 10^−16^
Positive regulation of cellular metabolic process	GO:0031325	53	2.93 × 10^−14^
Positive regulation of metabolic process	GO:0009893	56	5.93 × 10^−14^
Cell cycle G2/M phase transition	GO:0044839	18	7.84 × 10^−14^
G2/M transition of mitotic cell cycle	GO:0000086	17	2.86 × 10^−13^
Regulation of cellular metabolic process	GO:0031323	66	4.61 × 10^−13^
Response to endogenous stimulus	GO:0009719	34	8.64 × 10^−13^
Positive regulation of macromolecule metabolic process	GO:0010604	52	9.72 × 10^−13^
Positive regulation of nitrogen compound metabolic process	GO:0051173	49	1.62 × 10^−12^

**Table 3:** The top 15 pathways were specifically regulated by GTE’s preventive administration in obese zebrafish (*p* < 0.05).

Pathway	*p*-Value
Transcriptional regulation by STAT	3.68 × 10^−16^
Transcriptional regulation by CEBP	7.61 × 10^−12^
PAK signaling pathway	8.24 × 10^−12^
MAPK signaling pathway	2.03 × 10^−11^
PIN1 signaling pathway	2.51 × 10^−11^
JNK signaling pathway	8.28 × 10^−11^
Nucleophosmin signaling pathway	1.52 × 10^−10^
Transcriptional regulation by POU domain factor	1.16 × 10^−9^
Sirtuin signaling pathway	1.53 × 10^−9^
PARP signaling pathway	7.57 × 10^−9^
GH signaling pathway	8.46 × 10^−9^
Transcriptional regulation by SMAD	1.16 × 10^−8^
Transcriptional regulation by high-mobility group protein	1.40 × 10^−8^
Bcl-2 family signaling pathway	1.76 × 10^−8^
Arrestin signaling pathway	1.90 × 10^−8^

## Data Availability

The data presented in this study are available on request from the corresponding author.

## Supplementary Materials

The following are available online, Figure S1: Chromatograms of HPLC analysis of GTE, Table S1: Differentially expressed genes in preventive GTE-treated zebrafish compared with OF zebrafish, Table S2: GO function annotation results of 1115 differentially expressed genes by DAVID, Table S3: Primer sequences used in this study.

## Sample Availability

Green tea extract is available from the authors.

## References

[1] WHO (2020). Obesity and Overweight. https://www.who.int/en/news-room/fact-sheets/detail/obesity-and-overweight.

[2] Apovian C.M., Mechanick J.I. (2013). Obesity IS a disease!. Curr. Opin. Endocrinol. Diabetes Obes..

[3] Fu C., Jiang Y., Guo J., Su Z. (2016). Natural Products with Anti-obesity Effects and Different Mechanisms of Action. J. Agric. Food Chem..

[4] Hiramitsu M., Shimada Y., Kuroyanagi J., Inoue T., Katagiri T., Zang L., Nishimura Y., Nishimura N., Tanaka T. (2014). Eriocitrin ameliorates diet-induced hepatic steatosis with activation of mitochondrial biogenesis. Sci. Rep..

[5] Nakayama H., Shimada Y., Zang L., Terasawa M., Nishiura K., Matsuda K., Toombs C., Langdon C., Nishimura N. (2018). Novel Anti-Obesity Properties of *Palmaria mollis* in Zebrafish and Mouse Models. Nutrients.

[6] Karu N., Reifen R., Kerem Z. (2007). Weight gain reduction in mice fed Panax ginseng saponin, a pancreatic lipase inhibitor. J. Agric. Food Chem..

[7] Arai T., Kim H.-J., Chiba H., Matsumoto A. (2009). Anti-obesity effect of fish oil and fish oil-fenofibrate combination in female KK mice. J. Atheroscler. Thromb..

[8] Lyznicki J.M., Young D.C., Riggs J.A., Davis R.M. (2001). Obesity: Assessment and management in primary care. Am. Fam. Physician.

[9] Yamamoto S., Tsumura N., Nakaguchi T., Namiki T., Kasahara Y., Terasawa K., Miyake Y. (2011). Regional image analysis of the tongue color spectrum. Int. J. Comput. Assist. Radiol. Surg..

[10] Park S., Hahm K.-B., Oh T.-Y., Jin J.-H., Choue R. (2004). Preventive Effect of the Flavonoid, Wogonin, Against Ethanol-Induced Gastric Mucosal Damage in Rats. Dig. Dis. Sci..

[11] Uto N.S., Amitani H., Atobe Y., Sameshima Y., Sakaki M., Rokot N., Ataka K., Amitani M., Inui A. (2018). Herbal Medicine Ninjin’yoeito in the Treatment of Sarcopenia and Frailty. Front. Nutr..

[12] Koizumi K., Oku M., Hayashi S., Inujima A., Shibahara N., Chen L., Igarashi Y., Tobe K., Saito S., Kadowaki M. (2020). Suppression of Dynamical Network Biomarker Signals at the Predisease State (Mibyou) before Metabolic Syndrome in Mice by a Traditional Japanese Medicine (Kampo Formula) Bofutsushosan. Evid. Based Complement. Altern. Med..

[13] Perva-Uzunalić A., Škerget M., Knez Ž., Weinreich B., Otto F., Grüner S. (2006). Extraction of active ingredients from green tea (*Camellia sinensis*): Extraction efficiency of major catechins and caffeine. Food Chem..

[14] Prasanth M.I., Sivamaruthi B.S., Chaiyasut C., Tencomnao T. (2019). A Review of the Role of Green Tea (Camellia sinensis) in Antiphotoaging, Stress Resistance, Neuroprotection, and Autophagy. Nutrients.

[15] Bailey H.H., Mukhtar H. (2013). Green tea polyphenols and cancer chemoprevention of genitourinary cancer. Am. Soc. Clin. Oncol. Educ. Book.

[16] Saleem M., Adhami V.M., Siddiqui I.A., Mukhtar H. (2003). Tea Beverage in Chemoprevention of Prostate Cancer: A Mini-Review. Nutr. Cancer.

[17] Yang C.S., Wang H. (2016). Cancer Preventive Activities of Tea Catechins. Molecules.

[18] Legeay S., Rodier M., Fillon L., Faure S., Clere N. (2015). Epigallocatechin Gallate: A Review of Its Beneficial Properties to Prevent Metabolic Syndrome. Nutrients.

[19] Thielecke F., Boschmann M. (2009). The potential role of green tea catechins in the prevention of the metabolic syndrome—A review. Phytochemistry.

[20] Suzuki T., Pervin M., Goto S., Isemura M., Nakamura Y. (2016). Beneficial Effects of Tea and the Green Tea Catechin Epigallocatechin-3-gallate on Obesity. Molecules.

[21] Kaihatsu K., Yamabe M., Ebara Y. (2018). Antiviral Mechanism of Action of Epigallocatechin-3-O-gallate and Its Fatty Acid Esters. Molecules.

[22] Furushima D., Ide K., Yamada H. (2018). Effect of Tea Catechins on Influenza Infection and the Common Cold with a Focus on Epidemiological/Clinical Studies. Molecules.

[23] Tijburg L.B.M., Mattern T., Folts J.D., Weisgerber U.M., Katan M.B. (1997). Tea flavonoids and cardiovascular diseases: A review. Crit. Rev. Food Sci. Nutr..

[24] Pervin M., Unno K., Ohishi T., Tanabe H., Miyoshi N., Nakamura Y. (2018). Beneficial Effects of Green Tea Catechins on Neurodegenerative Diseases. Molecules.

[25] Zang L., Shimada Y., Nakayama H., Kim Y., Chu D.-C., Juneja L.R., Kuroyanagi J., Nishimura N. (2019). RNA-seq Based Transcriptome Analysis of the Anti-Obesity Effect of Green Tea Extract Using Zebrafish Obesity Models. Molecules.

[26] Kajimoto O., Kajimoto Y., Yabune M., Nakamura T., Kotani K., Suzuki Y., Nozawa A., Nagata K., Unno T., Sagesaka Y.M. (2005). Tea Catechins with a Galloyl Moiety Reduce Body Weight and Fat. J. Health Sci..

[27] Nagao T., Hase T., Tokimitsu I. (2007). A Green Tea Extract High in Catechins Reduces Body Fat and Cardiovascular Risks in Humans. Obesity.

[28] Auvichayapat P., Prapochanung M., Tunkamnerdthai O., Sripanidkulchai B.-O., Auvichayapat N., Thinkhamrop B., Kunhasura S., Wongpratoom S., Sinawat S., Hongprapas P. (2008). Effectiveness of green tea on weight reduction in obese Thais: A randomized, controlled trial. Physiol. Behav..

[29] Tingaud-Sequeira A., Ouadah N., Babin P.J. (2011). Zebrafish obesogenic test: A tool for screening molecules that target adiposity. J. Lipid Res..

[30] Huang D.W., Sherman B.T., Lempicki R.A. (2009). Systematic and integrative analysis of large gene lists using DAVID bioinformatics resources. Nat. Protoc..

[31] Khan N., Mukhtar H. (2018). Tea Polyphenols in Promotion of Human Health. Nutrients.

[32] Saeki K., Hayakawa S., Nakano S., Ito S., Oishi Y., Suzuki Y., Isemura M. (2018). In Vitro and In Silico Studies of the Molecular Interactions of Epigallocatechin-3-O-gallate (EGCG) with Proteins That Explain the Health Benefits of Green Tea. Molecules.

[33] Chen X.-Q., Hu T., Han Y., Huang W., Yuan H.-B., Zhang Y.-T., Du Y., Jiang Y.-W. (2016). Preventive Effects of Catechins on Cardiovascular Disease. Molecules.

[34] Pervin M., Unno K., Nakagawa A., Takahashi Y., Iguchi K., Yamamoto H., Hoshino M., Hara A., Takagaki A., Nanjo F. (2017). Blood brain barrier permeability of (−)-epigallocatechin gallate, its proliferation-enhancing activity of human neuroblastoma SH-SY5Y cells, and its preventive effect on age-related cognitive dysfunction in mice. Biochem. Biophys. Rep..

[35] Spiegelman B.M., Flier J.S. (2001). Obesity and the Regulation of Energy Balance. Cell.

[36] Osborn O., Olefsky J.M. (2012). The cellular and signaling networks linking the immune system and metabolism in disease. Nat. Med..

[37] Berlingieri M.T., Pallante P., Guida M., Nappi C., Masciullo V., Scambia G., Ferraro A., Leone V., Sboner A., Barbareschi M. (2007). UbcH10 expression may be a useful tool in the prognosis of ovarian carcinomas. Oncogene.

[38] Troncone G., Guerriero E., Pallante P., Berlingieri M.T., Ferraro A., Del Vecchio L., Gorrese M., Mariotti E., Iaccarino A., Palmieri E.A. (2009). UbcH10 expression in human lymphomas. Histopathology.

[39] Fajas L. (2013). Re-thinking cell cycle regulators: The cross-talk with metabolism. Front. Oncol..

[40] Richard A.J., Stephens J.M. (2011). Emerging roles of JAK–STAT signaling pathways in adipocytes. Trends Endocrinol. Metab..

[41] Gurzov E.N., Stanley W.J.W., Pappas E.G.E., Thomas H.H., Gough D.J. (2016). The JAK/STAT pathway in obesity and diabetes. FEBS J..

[42] Cernkovich E.R., Deng J., Bond M.C., Combs T.P., Harp J.B. (2008). Adipose-specific disruption of signal transducer and activator of transcription 3 increases body weight and adiposity. Endocrinology.

[43] Hong F., Radaeva S., Pan H.N., Tian Z., Veech R., Gao B. (2004). Interleukin 6 alleviates hepatic steatosis and ischemia/reperfusion injury in mice with fatty liver disease. Hepatology.

[44] Ki S.H., Park O., Zheng M., Morales-Ibanez O., Kolls J.K., Bataller R., Gao B. (2010). Interleukin-22 treatment ameliorates alcoholic liver injury in a murine model of chronic-binge ethanol feeding: Role of signal transducer and activator of transcription 3. Hepatology.

[45] Lefterova M.I., Lazar M.A. (2009). New developments in adipogenesis. Trends Endocrinol. Metab..

[46] Moseti D., Regassa A., Kim W.-K. (2016). Molecular Regulation of Adipogenesis and Potential Anti-Adipogenic Bioactive Molecules. Int. J. Mol. Sci..

[47] Jiang Y., Ding S., Li F., Zhang C., Sun-Waterhouse D., Chen Y., Li D. (2019). Effects of (+)-catechin on the differentiation and lipid metabolism of 3T3-L1 adipocytes. J. Funct. Foods.

[48] Wang S., Moustaid-Moussa N., Chen L., Mo H., Shastri A., Su R., Bapat P., Kwun I., Shen C.-L. (2014). Novel insights of dietary polyphenols and obesity. J. Nutr. Biochem..

[49] Wu B.-T., Hung P.-F., Chen H.-C., Huang R.-N., Chang H.-H., Kao Y.-H. (2005). The Apoptotic Effect of Green Tea (−)-Epigallocatechin Gallate on 3T3-L1 Preadipocytes Depends on the Cdk2 Pathway. J. Agric. Food Chem..

[50] Zang L., Morikane D., Shimada Y., Tanaka T., Nishimura N. (2011). A Novel Protocol for the Oral Administration of Test Chemicals to Adult Zebrafish. Zebrafish.

[51] Oka T., Nishimura Y., Zang L., Hirano M., Shimada Y., Wang Z., Umemoto N., Kuroyanagi J., Nishimura N., Tanaka T. (2010). Diet-induced obesity in zebrafish shares common pathophysiological pathways with mammalian obesity. BMC Physiol..

[52] Zang L., Shimada Y., Nishimura Y., Tanaka T., Nishimura N. (2013). A Novel, Reliable Method for Repeated Blood Collection from Aquarium Fish. Zebrafish.

[53] Zang L., Shimada Y., Nishimura Y., Tanaka T., Nishimura N. (2015). Repeated Blood Collection for Blood Tests in Adult Zebrafish. J. Vis. Exp..

[54] Peterson S.M., Freeman J.L. (2009). RNA Isolation from Embryonic Zebrafish and cDNA Synthesis for Gene Expression Analysis. J. Vis. Exp..

[55] Bullard J.H., Purdom E., Hansen K.D., Dudoit S. (2010). Evaluation of statistical methods for normalization and differential expression in mRNA-Seq experiments. BMC Bioinform..

[56] Sato H., Ishida S., Toda K., Matsuda R., Hayashi Y., Shigetaka M., Fukuda M., Wakamatsu Y., Itai A. (2005). New Approaches to Mechanism Analysis for Drug Discovery Using DNA Microarray Data Combined with KeyMolnet. Curr. Drug Discov. Technol..

