# Supplementary material for: Preventive Effects of Green Tea Extract against Obesity Development in Zebrafish
Source: Molecules. 2021 Apr 30;26(9):2627. doi: 10.3390/molecules26092627 (PMC8124760; doi:10.3390/molecules26092627)
Supplement: Supplementary file 1 [file molecules-26-02627-s001.zip › molecules-1160899-supplementary/Supplementary Files-revision/Supplementary_Figure_S1.pdf]

Supplementary Figure S1

Material and Methods

HPLC analysis

The green tea extract was dissolved in water to form a 10 mg/mL solution and then filtered. Analysis of the GTE was conducted using high-performance liquid chromatography (HPLC) on an HPLC analysis system (JASCO Corporation, Tokyo, Japan) under the following conditions: Develosil ODS-5 (Φ4.6 × 150 mm; Nomura Chemical, Seto, Japan); UV detector at 280 nm; mobile phase of A = H_2O and B = MeOH; gradient: A:B = 100:0, 30 min; A:B = 0:100, 10 min; A:B = 0:100, 10 min; A:B = 100:0, 10 min; injection volume 1 μL; flow rate 1 mL/min. (-)-Epigallocatechin (EGC), (-)-epigallocatechin gallate (EGCG), (-)-epicatechin (EC), and (-)-epicatechin gallate (ECG) were identified by LC-MS analysis.

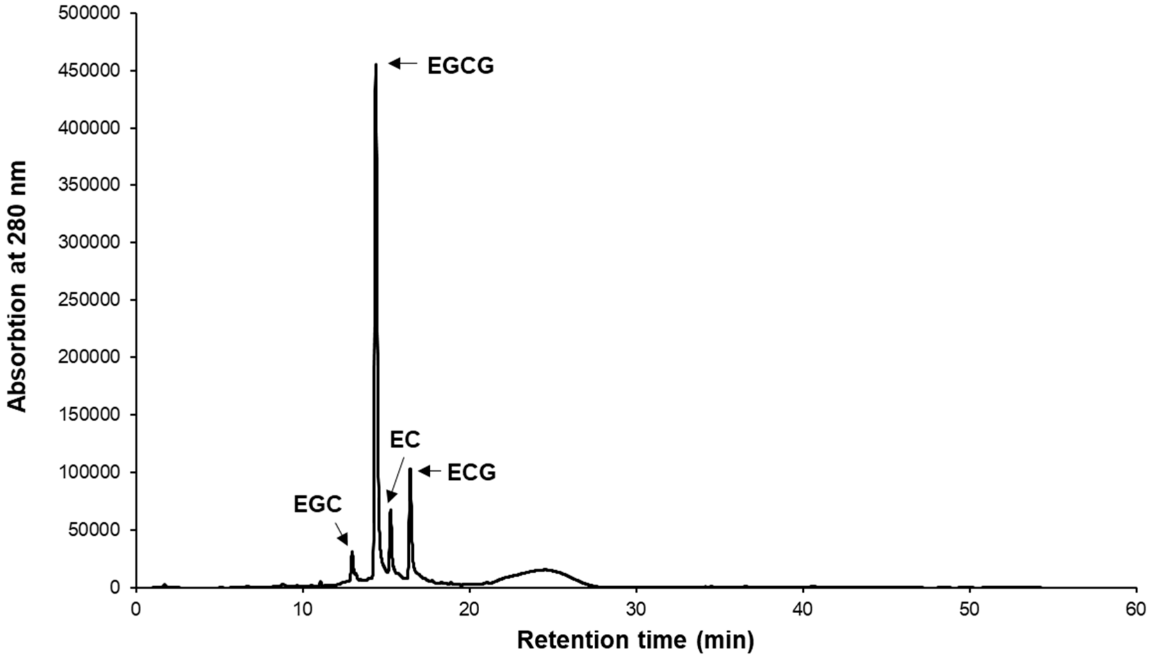

Figure S1. Chromatograms of HPLC analysis of GTE. The peaks were detected at UV 280 nm absorbance. (-)-Epigallocatechin (EGC), (-)-epigallocatechin gallate (EGCG), (-)-epicatechin (EC), (-)-epicatechin gallate (ECG).
